# TIC-XNet: a structured evidence translation framework for interpretable multimodal pediatric tic event detection with improved temporal alignment and fidelity

**DOI:** 10.3389/fpsyt.2026.1862470

**Published:** 2026-06-23

**Authors:** Liping Li, Jianping Wang, Kunying Zhou, Qian Li, Haoyu Wu, Huimin Song, Xiaoxia Fang

**Affiliations:** 1 Department of Pediatrics, Xinxiang Central Hospital, Xinxiang, Henan, China; 2 The Fourth Clinical College, Henan Medical University, Xinxiang, Henan, China; 3 School of Computer Science and Technology, Henan Institute of Science and Technology, Xinxiang, Henan, China; 4 The Second Affiliated Hospital, Xinxiang Medical University, Xinxiang, Henan, China

**Keywords:** evidence translation, explainable artificial intelligence, multimodal learning, pediatric tic disorders, tic disorder detection

## Abstract

**Objectives:**

This study aimed to develop an interpretable multimodal framework for detecting tic events in children with tic disorders by translating model decisions into structured, time-aligned evidence from synchronized video and physiological signals.

**Methods:**

TIC-XNet was developed to jointly analyze synchronized video, heart rate, and electrodermal activity signals and to generate structured evidence outputs. Recordings from 417 children with clinically diagnosed tic disorders were collected during structured clinical assessments and home-based observations. TIC-XNet was compared with a prediction-only black-box model and a *post-hoc* explainable model under matched predictive backbones and identical training settings.

**Results:**

Within the evaluated internal subject-level split, TIC-XNet achieved the best performance on the pooled shared test set, with a window-level AUC of 0.915 ± 0.019, higher event-level recall and precision, fewer missed events, and lower post-buffering prediction latency than the comparator models. Its translated outputs also showed higher decision fidelity, greater stability under perturbation, and closer temporal alignment with expert-annotated tic onsets. Subject-level translated numerical signals were associated with tic severity.

**Conclusions:**

These findings indicate that evidence translation can support more interpretable multimodal detection of tic events in children with tic disorders while remaining compatible with strong predictive performance.

## 1 Introduction

Pediatric tic disorders (TD) are neurodevelopmental conditions characterized by sudden, brief, and involuntary motor movements and vocalizations, encompassing a clinical spectrum that includes Tourette syndrome (1). Symptoms typically emerge in childhood and fluctuate substantially across time and contexts (2). Such variability is closely associated with emotional arousal, psychological stress, attentional state, and voluntary suppression, making tic expression difficult to capture reliably during brief clinical observation (3).

Current clinical evaluation of tic disorders mainly relies on physician observation and rating instruments such as the Yale Global Tic Severity Scale (YGTSS) (4). Although these approaches remain the clinical standard, they are constrained by subjectivity, limited observation duration, and reduced ecological validity (5). In practice, tic expression often varies across home, school, and clinic environments, and short in-clinic assessments may fail to reflect the temporal dynamics and contextual variability of symptoms (6). Objective and continuous monitoring approaches are therefore of considerable clinical value.

Objective assessment of tic disorders has therefore become an important research direction. Automated tic assessment has been investigated through video, computer vision, wearable sensing, and machine-learning methods. Existing studies have examined supervised video-based eye-tic quantification, video-derived Tourette syndrome diagnosis based on tic-related movements and temporal structure, machine-learning-based motor tic detection, and video-based tic-action evaluation using facial-region extraction and deep spatiotemporal feature learning (7–10). More broadly, automatic two-dimensional and three-dimensional video analysis with deep learning has become an important direction for neurological movement assessment (11). In addition to clinic-based video analysis, wearable sensor systems have been used for automatic tic characterization, while accessible video scenarios such as selfie-video and facial-video-based diagnosis have been explored to reduce monitoring burden and support less constrained tic assessment (12–14). Despite these advances, existing studies mainly focus on tic recognition, quantification, or diagnostic classification. Less attention has been paid to whether model decisions can be translated into structured and clinically reviewable evidence. Moreover, many current methods rely primarily on video, facial landmarks, or wearable motion signals alone, and therefore provide limited integration of observable tic behaviors with concurrent physiological context.

Multimodal behavioral and physiological learning provides a promising route for capturing both external behavior and internal autonomic responses. In affective computing and health-related monitoring, visual, vocal, and physiological signals have increasingly been integrated to improve robustness under noisy, incomplete, or context-dependent conditions (15, 16). Facial or video-based behavioral features have been jointly modeled with heart-related physiological signals, synchronized physiological streams, and occlusion-robust visual cues, supporting the value of coordinated behavioral–physiological modeling (17–19). Beyond visual–physiological fusion, recent studies have focused on dynamic alignment, incongruity-aware fusion, electrodermal activity, and context-sensitive physiological modeling, highlighting the importance of temporal coordination and signal reliability in multimodal learning (20–23). Nevertheless, most studies in this area focus on general emotion, stress, or arousal recognition rather than time-resolved tic-event detection. Existing approaches often emphasize classification performance, while the temporal correspondence between behavioral evidence and physiological dynamics is not always translated into clinically reviewable outputs.

Explainable artificial intelligence (XAI) has increasingly been promoted in healthcare as a means of improving transparency, accountability, and clinical trust (24, 25). In clinical decision support, explanations are expected to connect with clinical workflows and decision optimization rather than function only as additional visualization layers (26). Human-centered XAI studies further indicate that explanation interfaces should match users’ needs, tasks, and cognitive workflows (27, 28). At the same time, *post-hoc* explanations remain limited by concerns regarding stability, faithfulness, and suitability for time-resolved clinical event detection (29). These concerns are particularly relevant to physiological-signal analysis and healthcare monitoring, where interpretable event-level outputs are needed to clarify when an event occurs, what evidence supports it, and how different signals contribute to the decision (30, 31). Therefore, existing XAI methods may explain influential regions or signals, but they do not necessarily translate multimodal evidence into unified visual, numerical, temporal, and textual outputs for clinical review.

Taken together, existing studies have advanced automated tic detection, multimodal behavioral–physiological learning, and clinical XAI. However, important gaps remain for pediatric tic assessment. Existing tic-detection methods still primarily emphasize recognition, quantification, or diagnostic classification; multimodal behavioral–physiological studies rarely focus on time-resolved tic events; and clinical XAI methods are often evaluated in general diagnostic or imaging scenarios rather than in synchronized multimodal event-detection settings. These gaps suggest the need for a framework that can connect tic-event detection with multimodal evidence organization and clinically reviewable interpretation.

The main contributions of this study are summarized as follows:

1. We propose an interpretable multimodal framework for pediatric tic-event detection, which jointly models synchronized video, heart-rate, and EDA signals to capture both observable tic behaviors and accompanying physiological responses.
2. We design a structured evidence translation module that converts internal multimodal representations into coordinated visual, numerical, and textual evidence, enabling tic-detection decisions to be reviewed beyond prediction scores.
3. We introduce a temporal evidence alignment strategy to associate model-generated evidence with tic-event annotations and clinically meaningful time windows, improving the temporal traceability of detected tic events.
4. We establish a fairness-controlled evaluation protocol by comparing TIC-XNet with prediction-only and *post-hoc* explainable baselines under matched backbones, fusion settings, and subject-level splits.
5. We conduct comprehensive experiments on the MindTS-MMD cohort, demonstrating that TIC-XNet achieves competitive detection performance while producing more faithful, stable, and temporally aligned explanations associated with YGTSS severity.

The remainder of this paper is organized as follows. Section 2 describes the participants, multimodal data acquisition, annotation protocol, TIC-XNet framework, evidence translation mechanism, and evaluation strategy. Section 3 presents the experimental results, including prediction performance, explanation quality, ablation analysis, signal–severity associations, and qualitative evidence examples. Section 4 discusses the main findings, clinical implications, limitations, and future directions. Section 5 concludes the study.

## 2 Methods

### 2.1 Participants and dataset

Participants were recruited from Xinxiang Central Hospital and the Third Affiliated Hospital of Henan Medical University between June and September 2025 using a harmonized multimodal acquisition protocol. The final cohort comprised 417 children with clinically diagnosed tic disorders who completed synchronized video, heart rate (HR), and electrodermal activity (EDA) recordings during structured clinical assessments and home-based observations. Diagnoses were confirmed by qualified clinicians, and the resulting dataset was designated as MindTS-MMD.

The demographic, clinical, physiological, and recording characteristics of the cohort are summarized in **Table 1**. Briefly, the cohort included 417 participants with a mean age of 9.18 years and a mean YGTSS total score of 27.20. The dataset contained both clinical-center recordings and home recordings, with annotated tic events and tic-positive/tic-negative analysis windows constructed for model training and evaluation.

**Table 1:** Clinical and recording characteristics of the cohort.

Characteristic	Summary	Characteristic	Summary
Participants	417	Tic duration, years	2.67 (1.55)
Age, years	9.18 (1.83)	YGTSS total score	27.20 (6.43)
Female	73 (17.6%)	Clinical-center recordings	228
Male	344 (82.4%)	Home recordings	189
Total recording duration, hours	138.6	Mean heart rate, bpm	91.44 (10.03)
Annotated tic events	12,486	HRV, SDNN ms	45.46 (10.82)
Tic-positive windows	18,942	EDA tonic level, µS	4.68 (1.65)
Tic-negative windows	41,305	EDA phasic response frequency, per min	1.81 (0.73)

Continuous variables are reported as mean (standard deviation), and categorical variables are reported as counts and percentages. YGTSS, Yale Global Tic Severity Scale; HRV, heart rate variability; SDNN, standard deviation of normal-to-normal intervals; EDA, electrodermal activity.

Inclusion criteria were a confirmed tic disorder diagnosis, completed synchronized multimodal acquisition, and sufficient signal and video quality. Exclusion criteria included major neurological disorders, severe psychiatric comorbidities, unstable medication within 4 weeks before acquisition, and incomplete or low-quality recordings. Detailed preprocessing and window construction procedures are provided in **Supplementary Methods S1**.

To improve representation learning, self-supervised pretraining was additionally performed on public multimodal datasets from healthy subjects. These data were used only for representation learning and did not include tic-related labels or patient data from the present cohort. Details are provided in **Supplementary Methods S2**.

### 2.2 Multimodal data acquisition and annotation

Each recording included synchronized video, heart rate (HR), and electrodermal activity (EDA) acquired under a unified protocol. Video captured facial and upper-body behaviors, whereas HR and EDA reflected concurrent autonomic dynamics. Acquisition settings, including device configuration and sampling information, are summarized in **Supplementary Tables S1**, **S2**. HR-derived summary descriptors were computed within each 20 s analysis window and treated as short-window descriptors rather than standard short-term HRV indices.

Raw physiological signals were first preprocessed to reduce motion artifacts and short missing segments before multimodal alignment. EDA signals were low-pass filtered to suppress high-frequency noise. HR artifacts were corrected using local thresholding, and short corrupted or missing intervals were repaired using cubic spline interpolation. Windows with severe video loss, unrecoverable physiological artifacts, or incomplete timestamps were excluded according to predefined quality-control criteria.

Video, HR, and EDA streams were synchronized using timestamps recorded during the same acquisition session. All modalities were mapped to a unified temporal axis according to their absolute timestamps. Because video, HR, and EDA were acquired at different temporal resolutions, HR and EDA sequences were resampled onto a common temporal grid, while video streams were uniformly sampled or temporally pooled to the same number of temporal steps within each analysis window. This procedure ensured that the video representation, HR-related representation, and EDA representation at the same temporal index corresponded to the same relative time position.

After synchronization and preprocessing, multimodal streams were segmented into 20 s analysis windows with 10% overlap. Each window was represented as T aligned temporal steps for within-window evidence localization. A window was labeled tic-positive if it overlapped any annotated tic interval and tic-negative otherwise. Tic events were annotated offline by trained raters for onset, offset, and primary behavioral manifestation. These onset and offset timestamps were projected onto the same unified temporal axis used for multimodal alignment, ensuring that behavioral evidence, physiological dynamics, and tic-event labels were temporally comparable within each window.

Inter-rater reliability was assessed on a subset of independently annotated samples. Event-level annotation agreement was high (Cohen’s κ = 0.86), and temporal consistency of tic onset and offset annotation was also strong (ICC = 0.91). Detailed annotation procedures are provided in **Supplementary Methods S3**.

Overall, preprocessing included physiological artifact correction, EDA filtering, HR interpolation, timestamp-based multimodal synchronization, temporal resampling, 20 s window segmentation with 10% overlap, and tic-label projection onto aligned analysis windows. YGTSS scores were used only for association and stratified analyses, not as a prediction target. Predefined physiological descriptors were used as auxiliary inputs and retained for quantitative evidence reporting, but were not optimized as independent prediction targets.

### 2.3 Comparator models and fairness control

To quantify the added value of structured evidence translation, TIC-XNet was compared with two baseline models: a prediction-only black-box model (BB) and a *post-hoc* explainable model (PH-XAI). BB produced only window-level tic probabilities. PH-XAI used the same predictive backbone as BB and generated explanations at inference by applying Integrated Gradients to physiological signals and spatiotemporal Grad-CAM to video. TIC-XNet shared the same predictive backbone and multimodal input pipeline, but additionally translated internal decision evidence into structured visual, numerical, and textual outputs.

The shared predictive backbone consisted of modality-specific encoders, a multimodal fusion block, and a window-level prediction head. For each 20 s analysis window, the video branch used a 3D-ResNet-18 encoder to extract spatiotemporal visual features from uniformly sampled frame sequences. The HR and EDA branches used 1D-CNN + GRU encoders to model short-term physiological dynamics. The physiological GRU hidden size was 128, and the video and physiological feature dimensions were both projected to 256. Modality-specific features were concatenated into a 512-dimensional fused representation and passed to a two-layer MLP prediction head with 128 hidden units and sigmoid output for tic/non-tic classification. TIC-XNet further included evidence heads for structured translation, including a visual evidence head with 128 hidden units and a 64-dimensional output, and a physiological evidence head with 96 hidden units and a 32-dimensional output. Evidence vectors were L2-normalized before deterministic evidence translation.

To ensure a fair comparison, BB, PH-XAI, and TIC-XNet were matched in input representation, temporal windowing, backbone architecture, parameter scale, fusion strategy, training protocol, and evaluation procedure. All models used the same synchronized video, HR, and EDA inputs, 20 s analysis windows, modality-specific encoding pipelines, multimodal fusion block, and window-level prediction head. Training used the same subject-level partitions, weighted binary cross-entropy loss, AdamW optimizer, initial learning rate of $1\times 10^{-4}$, cosine annealing, batch size of 16, and up to 80 epochs, with early stopping based on validation loss. The probability threshold was selected on the validation set by maximizing window-level F1 and was then fixed for all held-out test evaluations.

Thus, the only intentional difference among the three model conditions was the explainability paradigm: BB provided prediction scores only, PH-XAI generated *post-hoc* attribution maps after prediction, and TIC-XNet produced structured evidence translation within the model output pathway. This design allowed performance and interpretability differences to be attributed primarily to the explanation and evidence-translation strategy rather than to differences in model capacity, data partitioning, or training configuration. Exact implementation details are provided in **Supplementary Methods S4** and **Supplementary Table 4**.

### 2.4 TIC-XNet framework

**Figure 1:**
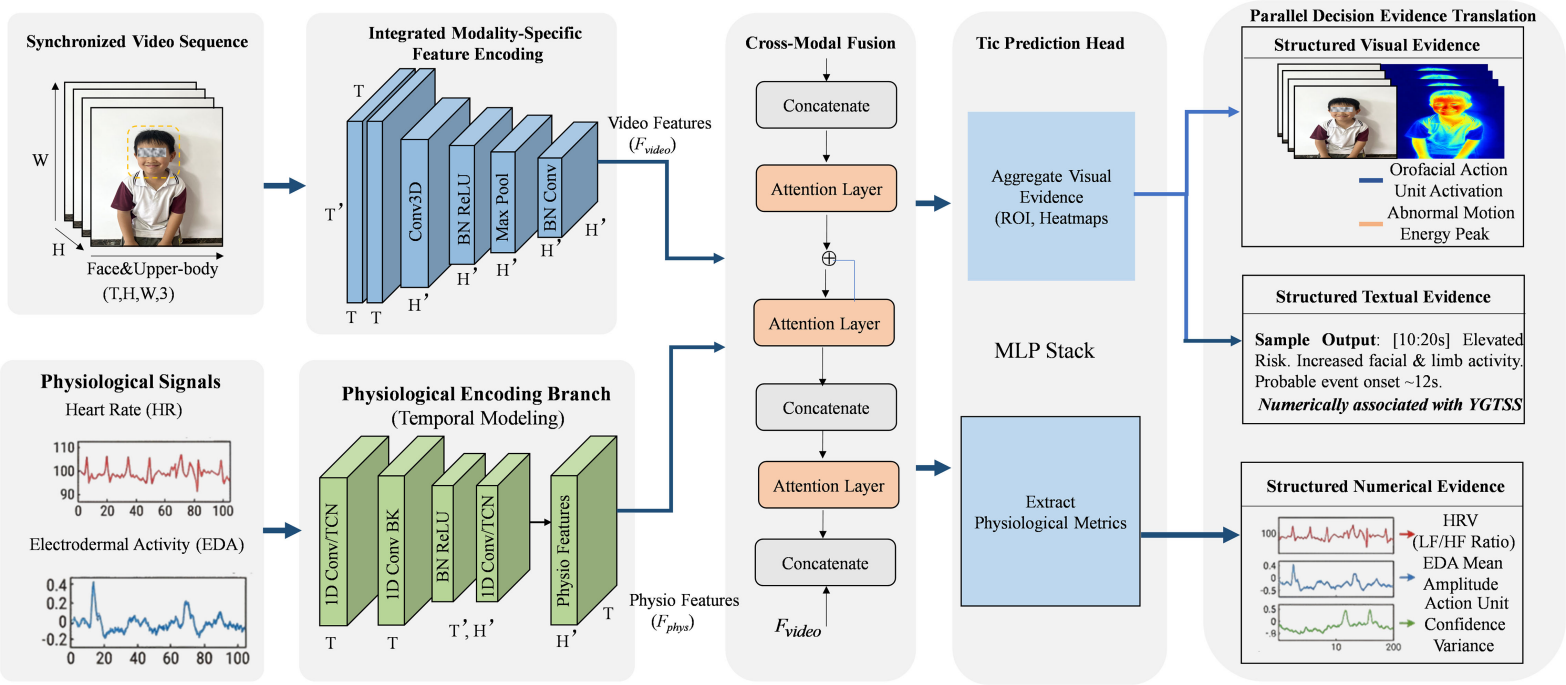
Overview of TIC-XNet.

TIC-XNet is a multimodal deep learning framework for pediatric tic assessment from synchronized video and physiological signals. For each fixed-length analysis window, the model jointly produces a window-level tic probability and temporally resolved evidence signals that are subsequently translated into structured multimodal outputs. As illustrated in **Figure 1**, the framework consists of four stages: temporal alignment and input representation, modality-specific encoding, multimodal fusion for tic prediction, and deterministic evidence extraction from the shared latent space. In this way, prediction and interpretability are generated within the same forward computation rather than through a separate *post-hoc* procedure.

For the i-th analysis window from subject n and recording session s, let $V_{i}$, $S_{i}^{h}$, and $S_{i}^{e}$ denote the temporally aligned video, HR-related sequence, and EDA sequence, respectively. All streams are resampled to the same number of aligned temporal steps T within each 20 s analysis window. Let $b_{i}$ denote the absolute start time of the window. The dominant fused evidence peak is identified as $t_{i}^{*}=argmax_{t\in \{1,\ldots ,T\}}a_{i}^{m}(t),$ and its back-projected absolute timestamp is defined as ${\hat{\tau }}_{i}=b_{i}+\frac{(t_{i}^{*}-1)L}{T-1},$ where L=20 s is the fixed window length.

To capture modality-specific dynamics, TIC-XNet uses separate encoding branches for video, HR-related signals, and EDA. The video branch applies a spatiotemporal encoder $f_{v}(\cdot )$ to extract motion- and appearance-aware features, as shown in Equation 1:

(1) $$\begin{array}{c} Z_{i}^{v}=f_{v}(V_{i}),\;Z_{i}^{v}\in {\mathbb{R}}^{T\times d_{v}}\\ {\tilde{Z}}_{i}^{h}=f_{h}(S_{i}^{h}),\;\;{\tilde{Z}}_{i}^{h}\in {\mathbb{R}}^{T\times d_{h}} \end{array}$$

In parallel, the predefined HR-derived summary vector $F_{i}^{h}$ is projected into a latent representation through a learnable mapping $\phi_{h}^{g}(\cdot )$, i.e., $g_{i}^{h}=\phi_{h}^{g}(F_{i}^{h}),\;g_{i}^{h}\in {\mathbb{R}}^{d_{g}}.\;$The projected summary representation is broadcast along the temporal axis and fused with the temporal HR representation to obtain the final HR-related latent feature $Z_{i}^{h}(t)=\phi_{h}([{\tilde{Z}}_{i}^{h}(t);g_{i}^{h}]),t=1,\ldots ,T,$ where [·;·] denotes feature concatenation and $\phi_{h}(\cdot )$ denotes a learnable projection.

The EDA branch models sympathetic activity dynamics as $Z_{i}^{e}=f_{e}(S_{i}^{e}),\;Z_{i}^{e}\in {\mathbb{R}}^{T\times d_{e}}.$ The HR-related and EDA representations are then fused into a shared physiological representation $Z_{i}^{p}(t)=\phi_{p}([Z_{i}^{h}(t);Z_{i}^{e}(t)]),\;\;Z_{i}^{p}\in {\mathbb{R}}^{T\times d_{p}},$ where $\phi_{p}(\cdot )$ denotes a learnable physiological fusion mapping. After modality-specific encoding, the aligned video and physiological representations are fused at each temporal step, as shown in Equation 2:

(2) $$Z_{i}^{m}(t)=\phi_{f}([Z_{i}^{v}(t);Z_{i}^{p}(t)[),\;t=1,\ldots ,T,$$

where $\phi_{f}(\cdot )$ denotes the multimodal fusion function. A window-level representation is obtained by temporal pooling, as shown in Equation 3:

(3) $${\bar{Z}}_{i}^{m}=\text{Pool}({\left\{Z_{i}^{m}(t)\right\}}_{t=1}^{T})$$

The pooled multimodal feature is then passed to the classification head, as shown in Equation 4:

(4) $$\begin{array}{c} s_{i}=W_{c}{\bar{Z}}_{i}^{m}+b_{c}\\ {\hat{y}}_{i}=\sigma (s_{i})=\frac{1}{1+e^{-s_{i}}} \end{array}$$

where $s_{i}$ is the tic-risk logit and ${\hat{y}}_{i}$ is the predicted tic probability for the i-th window.

In addition to risk prediction, TIC-XNet derives temporally resolved evidence directly from the same latent representations used for classification. Specifically, scalar evidence intensity scores are obtained by linear projections of the modality-specific and fused features, as shown in Equation 5:

(5) $$\begin{array}{c} a_{i}^{v}(t)=w_{v}^{⊤}Z_{i}^{v}(t)+b_{v}\\ a_{i}^{p}(t)=w_{p}^{⊤}Z_{i}^{p}(t)+b_{p}\\ a_{i}^{m}(t)=w_{m}^{⊤}Z_{i}^{m}(t)+b_{m} \end{array}$$

where $a_{i}^{v}(t)$, $a_{i}^{p}(t)$, and $a_{i}^{m}(t)$ denote the video, physiological, and fused evidence intensity at temporal step t, respectively. These evidence sequences are not generated by an auxiliary explanation branch; rather, they are computed from the same encoded representations that support tic prediction. The resulting evidence is subsequently translated into structured visual, numerical, and textual outputs, as described below.

For notation consistency, $S_{i}^{e}$ denotes the preprocessed EDA sequence, $Z_{i}^{m}(t)$ denotes the fused multimodal representation, $a_{i}^{k}(t)$ denotes the raw temporal evidence for modality k∈{v,p,m}, ${\tilde{a}}_{i}^{k}(t)$ denotes its normalized form, and $E_{i}$ denotes the translated output tuple.

### 2.5 Evidence translation, quantitative signal definition, and structured interpretability

A central component of TIC-XNet is the evidence translation module, which converts internal multimodal evidence into structured, time-aligned outputs for direct inspection and quantitative evaluation. Unlike *post-hoc* attribution methods, TIC-XNet derives translated outputs deterministically from the same modality-specific and fused latent representations used for tic detection, thereby keeping prediction and interpretation tightly coupled within a shared computational pathway.

Let $a_{i}^{v}(t)$, $a_{i}^{p}(t)$, and $a_{i}^{m}(t)$ denote the raw temporal evidence intensity derived from the video, physiological, and fused multimodal representations, respectively. To make evidence comparable across windows and modalities, each evidence sequence is normalized into a temporal importance profile, as shown in Equation 6:

(6) $${\tilde{a}}_{i}^{k}(t)=\frac{\exp(a_{i}^{k}(t))}{\sum_{\tau =1}^{T}\exp(a_{i}^{k}(\tau ))}$$

For visual interpretation, the video evidence is further decomposed into predefined anatomical regions R={face,upper limbs,trunk}. Let $g_{r}(\cdot )$ denote the region-specific scoring function for anatomical region r. The region-level visual evidence trajectory is defined as Equation 7

(7) $$\rho_{i}^{r}(t)=\frac{\exp(g_{r}(Z_{i}^{v}(t)))}{\sum_{r^{\prime }\in R}\exp(g_{r^{\prime }}(Z_{i}^{v}(t)))}$$

This formulation yields a time-varying estimate of the relative contribution of each anatomical region within the video branch.

For physiological interpretation, the shared physiological evidence is decomposed into HR-related and EDA-related support signals through scalar projections of the corresponding latent features, as shown in Equation 8:

(8) $$\begin{array}{c} e_{i}^{\text{HR}}(t)=w_{\text{HR}}^{⊤}Z_{i}^{h}(t)+b_{\text{HR}}\\ e_{i}^{\text{EDA}}(t)=w_{\text{EDA}}^{⊤}Z_{i}^{e}(t)+b_{\text{EDA}} \end{array}$$

These support signals are used only for translated output reporting and interpretability analysis; they are not optimized as independent prediction targets.

In addition to temporal evidence profiles, TIC-XNet reports translated numerical signals for subject-level association analysis. These descriptors are attached to the translated output rather than trained as separate supervision objectives. For the i-th analysis window, the physiological signals include a short-window spectral asymmetry index, defined as $q_{i}^{\text{SWSAI}}=\frac{LF_{i}}{HF_{i}+\epsilon }$, where $LF_{i}$ and $HF_{i}$ denote low- and high-frequency spectral components estimated from the synchronized HR/IBI sequence within the 20 s analysis window, and ϵ is a small positive constant for numerical stability. Because the window length was only 20 s, SWSAI was used only as a relative short-window descriptor of spectral asymmetry and should not be interpreted as a standard LF/HF ratio or canonical short-term HRV metric.

Let ${\bar{S}}_{i}^{e}(t)$ denote the preprocessed EDA amplitude sequence in the same window. The translated EDA signal is defined as $q_{i}^{\text{EDA}}=\frac{1}{T}\sum_{t=1}^{T}{\bar{S}}_{i}^{e}(t).$ For behavioral quantification, TIC-XNet derives three numerical descriptors from the video latent representation. Let $u_{i}(t)=g_{\text{AU}}(Z_{i}^{v}(t))$ denote the tic-related orofacial action-unit confidence estimated from the video branch, and let $m_{i}(t)$ denote the corresponding motion-energy trace defined as Equation 9:

(9) $$m_{i}(t)=\{\begin{array}{cc} 0, & t=1,\\ g_{M}(Z_{i}^{v}(t))-g_{M}{\left(Z_{i}^{v}(t-1)\right)}_{2}, & t=2,\ldots ,T, \end{array}$$

where $g_{\text{AU}}(\cdot )$ and $g_{M}(\cdot )$ are learnable projections from the shared video representation. The translated behavioral signals are then defined as Equation 10:

(10) $$\begin{array}{c} q_{i}^{\text{AUV}}={\text{Var}}_{t}(u_{i}(t))\\ q_{i}^{\text{ME}}=max_{t}m_{i}(t)\\ q_{i}^{\text{OFA}}=\frac{1}{T}\sum_{t=1}^{T}u_{i}(t) \end{array}$$

Here, $q_{i}^{\text{AUV}}$ denotes tic action unit confidence variance, $q_{i}^{\text{ME}}$ denotes abnormal motion energy peak, and $q_{i}^{\text{OFA}}$ denotes mean orofacial action-unit activation strength.

For the signal–severity analyses, each translated numerical signal is summarized at the subject level as the median across analysis windows overlapping expert-annotated tic intervals $Q_{n}^{\left(k\right)}={\text{median}}_{i\in W_{n}^{+}}q_{i}^{\left(k\right)},$ where $W_{n}^{+}$ denotes the set of tic-positive windows for subject n, and k indexes the translated numerical signal.

Accordingly, the translated output for analysis window i is represented as $\;E_{i}=\{{\hat{y}}_{i},{\tilde{a}}_{i}^{m}(t),{\left\{\rho_{i}^{r}(t)\right\}}_{r\in R},e_{i}^{\text{HR}}(t),e_{i}^{\text{EDA}}(t),q_{i},T_{i}\},$ where $q_{i}=[q_{i}^{\text{SWSAI}},q_{i}^{\text{EDA}},q_{i}^{\text{AUV}},q_{i}^{\text{ME}},q_{i}^{\text{OFA}}]$ is the translated numerical signal vector, and $T_{i}$ is the deterministic textual summary.

To ensure transparency and to avoid hallucination risk, $T_{i}$ is generated using fixed rule-based templates rather than a generative language model. The textual output follows a predefined slot structure: [Temporal Focus] + [Behavioral Phenotype] + [Physiological Context]. Temporal focus is determined by the dominant fused evidence peak $t_{i}^{*}$, behavioral phenotype by the dominant region-level visual evidence $\rho_{i}^{r}(t)$, and physiological context by threshold rules relative to participant-session-specific baseline statistics estimated from tic-negative windows within the same recording session. The complete lookup rules are provided in **Supplementary Table 5**.

Accordingly, evidence translation in TIC-XNet is implemented as a deterministic reorganization of temporally aligned multimodal evidence derived from the same latent representations used for tic detection. The resulting translated outputs are directly amenable to fidelity analysis, perturbation testing, temporal alignment evaluation, and signal–severity association analysis.

### 2.6 Performance evaluation and statistical analysis

All experiments were conducted using subject-level data partitioning to prevent information leakage across analysis windows from the same participant. The full MindTS-MMD cohort was divided into training, validation, and test sets at a ratio of 80%:10%:10%, with stratification to preserve tic prevalence and recording scenario distribution. The same subject-level split was used across five independent training runs with different random seeds. Unless otherwise stated, comparative model-performance metrics are reported as mean ± standard deviation across the five runs. When clinically interpretable pooled event counts are additionally reported, they are presented as descriptive summaries on the shared held-out test partition.

#### 2.6.1 Window-level and event-level performance

Window-level classification performance was evaluated using ROC-AUC, recall, precision, and F1 score. Precision, recall, and F1 score are defined as Equation 11:


$$\text{Precision}=\frac{TP}{TP+FP}$$

$$\text{Recall}=\frac{TP}{TP+FN}$$

(11) $$F1=\frac{2\cdot \text{Precision}\cdot \text{Recall}}{\text{Precision}+\text{Recall}}$$

where TP, FP, and FN denote true positives, false positives, and false negatives, respectively. The probability threshold used to binarize window-level predictions was selected on the validation set by maximizing window-level F1 and was then fixed for all held-out test evaluations.

Event-level predictions were obtained by merging consecutive tic-positive windows using a minimum-gap rule of 0.5 s. A predicted event was considered correct if it overlapped an expert-annotated tic interval. Event-level recall, event-level precision, and missed-event counts were computed from the resulting merged predictions.

Post-buffering prediction latency was defined as the wall-clock time required to generate a window-level prediction after the current 20 s analysis window had been fully buffered. For fairness, this metric quantified predictive forward-pass latency only. For PH-XAI, *post-hoc* attribution generation was not included in the latency calculation.

#### 2.6.2 Explainability evaluation

Explainability was evaluated in terms of decision fidelity, stability under perturbation, and temporal alignment with expert annotations.

Decision fidelity was quantified by masking the top m% of model-identified temporal evidence and measuring the resulting change in predicted tic probability, as shown in Equation 12:

(12) $$\Delta P_{m}={\hat{y}}_{i}^{\left(m\right)}-{\hat{y}}_{i}$$

where ${\hat{y}}_{i}$ is the original predicted probability and ${\hat{y}}_{i}^{\left(m\right)}$ is the probability after masking the top m% evidence. More negative $\Delta P_{m}$ values indicate stronger causal relevance of the masked evidence. Fidelity was evaluated at masking ratios of 10%, 25%, and 50%, and summarized by the normalized area under the $\Delta P_{m}$-masking curve (Fidelity AUC).

Stability under perturbation was assessed using Jensen–Shannon (JS) divergence between normalized evidence distributions before and after controlled perturbations. Two perturbation types were considered: temporal shifts and additive Gaussian noise. Lower JS divergence indicates greater explanation stability.

Temporal alignment was evaluated by comparing the back-projected timestamp of the dominant fused evidence peak, ${\hat{\tau }}_{i}$, with the expert-annotated tic onset $\tau_{i}^{\text{gt}}$ for windows overlapping annotated tic events. Alignment quality was summarized by the mean absolute onset offset, the signed peak-to-onset offset, and the alignment success rate within ±100 ms.

#### 2.6.3 Statistical analysis

Continuous variables are reported as mean ± standard deviation unless otherwise specified. Pairwise comparisons between TIC-XNet and comparator models (BB and PH-XAI) were performed using two-sided Wilcoxon signed-rank tests on run-level paired metrics obtained under identical subject-level splits. A p-value <0.05 was considered statistically significant.

For the signal–severity analyses, Pearson and Spearman correlation coefficients were computed using subject-level translated numerical signals and YGTSS total scores. Corresponding 95% confidence intervals are reported. Multiple testing was controlled using false discovery rate (FDR) correction.

For severity-group analyses, participants were divided into mild, moderate, and severe groups according to YGTSS total score. Overall group differences were assessed using the Kruskal–Wallis test, and monotonic trends across ordered severity strata were evaluated using the Jonckheere–Terpstra test. Because the primary purpose of this analysis was to evaluate ordered severity-related trends rather than pairwise group differences, no *post-hoc* pairwise comparisons were performed. Therefore, conclusions from the severity-group analysis were restricted to overall group differences and monotonic trend patterns. Effect sizes are reported as η² where appropriate.

## 3 Results

### 3.1 Prediction performance

To evaluate whether structured evidence translation compromised predictive capacity, TIC-XNet was compared with a prediction-only black-box model (BB) and a *post-hoc* explainable model (PH-XAI) under matched predictive backbones, identical training settings, and identical subject-level partitions. **Table 2** summarizes the scenario-specific results obtained from structured clinical recordings and naturalistic home recordings.

**Table 2:** Scenario-specific window-level and event-level prediction performance across recording settings.

Model	Setting	ROC-AUC (mean ± SD)	Window-level recall (mean ± SD)	Window-level F1 (mean ± SD)	Event recall (%)	Event precision (%)	Post-buffering prediction latency (ms, mean ± SD)	False negatives (count)
BB	Clinical	0.908 ± 0.018	0.842 ± 0.028	0.828 ± 0.026	80.2	76.8	118 ± 40	18
	Home	0.874 ± 0.021	0.810 ± 0.031	0.796 ± 0.029	76.4	73.5	132 ± 44	24
PH-XAI	Clinical	0.918 ± 0.017	0.870 ± 0.024	0.854 ± 0.023	85.2	81.0	95 ± 36	13
	Home	0.886 ± 0.020	0.838 ± 0.028	0.822 ± 0.026	82.2	78.1	101 ± 40	16
TIC-XNet	Clinical	0.930 ± 0.016	0.882 ± 0.022	0.863 ± 0.021	87.0	83.4	82 ± 34	11
	Home	0.902 ± 0.019	0.854 ± 0.027	0.837 ± 0.025	84.5	79.3	88 ± 36	13

ROC-AUC, window-level recall, window-level F1, and post-buffering prediction latency are reported as mean ± standard deviation over five independent runs. Post-buffering prediction latency denotes the computational wall-clock time required to generate a window-level prediction after the current 20 s analysis window has been fully buffered. For PH-XAI, post-hoc attribution generation time was not included in the latency calculation. Event recall, event precision, and false negatives are reported as scenario-specific descriptive summaries computed on the shared held-out evaluation set.

At the window level, within the evaluated internal test cohort, TIC-XNet achieved the highest ROC-AUC, recall, and F1 score in both settings, indicating that deterministic evidence translation remained compatible with strong predictive discrimination under the current evaluation setting. This advantage extended to the event level, where TIC-XNet yielded the highest event recall and event precision while minimizing missed tic events relative to both comparator models.

TIC-XNet further showed the lowest post-buffering prediction latency in both settings, suggesting favorable computational efficiency once the current analysis window had been fully buffered. Although all models showed reduced performance in home recordings relative to structured clinical recordings, the degradation was less pronounced for TIC-XNet. In particular, within the evaluated home-recording subset, the model maintained an ROC-AUC above 0.90 and an event recall of 84.5%, suggesting relatively stable performance under increased noise and contextual variability.

### 3.2 Explainability and evidence translation effectiveness

After confirming predictive performance, we next evaluated explanation quality in terms of decision fidelity, stability under perturbation, and temporal alignment with expert annotations. As shown in **Table 3**, TIC-XNet consistently outperformed the *post-hoc* explainable model (PH-XAI) across all three aspects.

**Table 3:** Quantitative evaluation of explanation quality for TIC-XNet and PH-XAI.

Evaluation aspect	Metric	TIC-XNet	PH-XAI
Decision fidelity	ΔP after masking top 10% evidence	−0.52 ± 0.11	−0.38 ± 0.14
	ΔP after masking top 25% evidence	−0.78 ± 0.09	−0.65 ± 0.12
	ΔP after masking top 50% evidence	−0.95 ± 0.04	−0.88 ± 0.08
	Fidelity AUC	0.92	0.85
Stability under perturbation	JS divergence (± 50 ms shift)	0.08 ± 0.03	0.15 ± 0.05
	JS divergence (± 100 ms shift)	0.12 ± 0.04	0.24 ± 0.07
	JS divergence (Gaussian noise, SNR 20 dB)	0.05 ± 0.02	0.09 ± 0.04
	JS divergence (Gaussian noise, SNR 10 dB)	0.11 ± 0.03	0.21 ± 0.06
Temporal alignment	Mean absolute onset offset (ms)	42 ± 28	68 ± 41
	Alignment success rate (≤100 ms)	89.7%	73.4%
	Signed peak-to-onset offset (ms)	−15 ± 22	35 ± 50

ΔP denotes the change in predicted tic probability after masking model-identified evidence. Fidelity AUC denotes the normalized area under the ΔP–mask ratio curve. Stability was quantified using Jensen–Shannon (JS) divergence between explanation distributions before and after perturbation. Temporal alignment metrics were computed from the back-projected timestamps of within-window fused evidence peaks selected from analysis windows overlapping expert-annotated tic events, rather than from merged window boundaries. Fidelity AUC and alignment success rate are reported as pooled descriptive values on the shared held-out evaluation set.

For decision fidelity, masking model-identified evidence led to larger probability reductions in TIC-XNet than in PH-XAI across all masking ratios, indicating that the translated evidence was more tightly coupled to decision-critical features. TIC-XNet also achieved a higher fidelity AUC, further supporting that its evidence representation more faithfully reflected the basis of the model prediction.

For stability, TIC-XNet showed consistently lower Jensen–Shannon divergence under both temporal shifts and additive noise, indicating greater robustness of the translated evidence to perturbation. In addition, TIC-XNet achieved better temporal alignment with expert annotations, as reflected by smaller onset offsets and a higher alignment success rate. Notably, these localization metrics were computed from back-projected within-window fused evidence peaks rather than from coarse window boundaries, indicating that the translated evidence retained fine-grained temporal information beyond the window-level prediction itself.

Together, these results indicate that evidence translation yields explanations that are more faithful, more stable, and more temporally precise than conventional *post-hoc* attribution.

### 3.3 Ablation study of evidence translation

An ablation study was conducted to assess the contributions of multimodal integration and the key design components of the evidence translation framework. **Table 4** summarizes the pooled results obtained on the shared held-out test set, combining samples from both the structured clinical recordings and the naturalistic home recordings. The removal of either complementary modalities or structured translation components led to consistent declines in both detection performance and explanation quality.

**Table 4:** Ablation study of evidence translation in TIC-XNet using pooled results on the shared held-out test set.

Model variant	ROC-AUC	Window recall	Window F1	Event recall	Event precision	Post-buffering prediction latency (ms)	Fidelity AUC	Mean JSD	Alignment success (%)
PH-XAI (reference)	0.902	0.854	0.838	83.7	79.5	98	0.85	0.19	73.4
Physiological-only TIC-XNet	0.758	0.685	0.642	61.4	52.8	245	0.80	0.28	42.5
Video-only TIC-XNet	0.892	0.842	0.825	81.5	77.2	105	0.82	0.22	78.4
w/o evidence translation	0.904	0.857	0.840	83.9	79.9	95	0.87	0.17	76.1
w/o temporal alignment	0.907	0.861	0.842	84.1	80.3	91	0.88	0.15	78.5
w/o structured evidence	0.910	0.864	0.845	84.7	80.8	89	0.89	0.13	81.2
TIC-XNet (full)	0.915	0.868	0.847	85.4	81.2	85	0.92	0.11	86.9

ROC-AUC, window recall, window F1, post-buffering prediction latency, Fidelity AUC, and Mean JSD are reported as mean values across five independent runs on the shared held-out test set. Post-buffering prediction latency denotes the computational wall-clock time required to generate a window-level prediction after the current 20 s analysis window had been fully buffered. For PH-XAI, post-hoc attribution generation time was not included in the latency calculation. Alignment success is defined as the percentage of dominant fused evidence peaks occurring within ± 100 ms of expert-annotated tic onsets. Improvements of TIC-XNet over PH-XAI were assessed using paired run-level comparisons under identical subject-level splits, with statistical significance defined as p<0.05.

To examine the role of multimodal integration, the full TIC-XNet was first compared with single-modality variants. The physiological-only model showed the weakest performance, with substantial reductions in ROC-AUC, window-level F1, and event-level recall, together with poor temporal alignment (Alignment success = 42.5%). It also showed higher post-buffering prediction latency (245 ms) than the full model. This pattern suggests that physiological signals alone did not provide sufficient temporal specificity for accurate tic localization, while also offering no computational advantage under the present implementation setting. By contrast, the video-only model retained relatively strong predictive performance (ROC-AUC = 0.892), indicating that overt motor behavior carried substantial information for tic detection. However, its explanation quality remained clearly inferior to that of the full model, with lower Fidelity AUC (0.82 vs. 0.92) and reduced alignment success (78.4% vs. 86.9%). Taken together, these results suggest that visual information primarily supports temporal localization and behavioral characterization, whereas synchronized physiological signals provide complementary internal context that improves the stability and coherence of the translated evidence.

**Figure 2:**
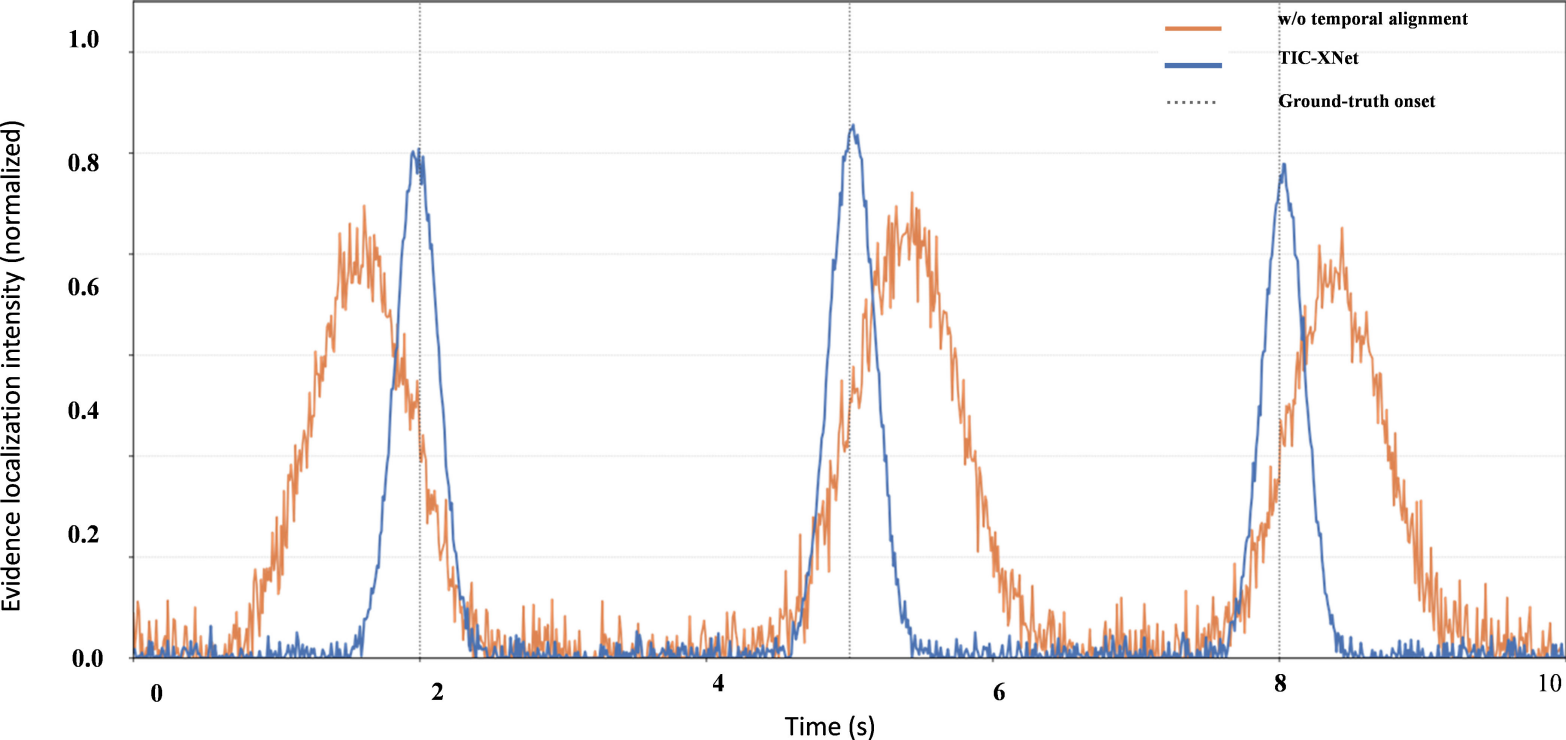
Effect of temporal alignment on evidence localization. Evidence peaks are more concentrated and better aligned with expert-annotated tic onsets when temporal alignment is applied.

We next evaluated the specific contribution of the translation-related design components. Removing the evidence translation module reduced event-level recall and precision and was also associated with higher post-buffering prediction latency relative to the full model. At the explanation level, removing structured evidence outputs reduced decision fidelity and increased perturbation divergence (Mean JSD = 0.13 vs. 0.11), suggesting a weaker coupling between the reported evidence and the underlying decision process. Likewise, removing temporal alignment reduced alignment success from 86.9% to 78.5%, with explanation peaks deviating further from expert-annotated tic onsets. As illustrated in **Figure 2**, the absence of temporal alignment resulted in more dispersed evidence peaks and weaker correspondence with annotated events, whereas the full TIC-XNet preserved more temporally concentrated and clinically interpretable evidence localization.

Across all evaluated metrics, the full TIC-XNet achieved the highest Fidelity AUC, the lowest perturbation divergence, and the strongest temporal alignment while preserving the best predictive performance. These findings suggest that the observed gain was unlikely to be attributable to the shared predictive backbone alone and was instead associated with the joint contribution of multimodal verification and structured evidence translation.

### 3.4 Signal–severity association analysis

To examine whether the translated numerical outputs captured clinically meaningful variation beyond detection performance, we evaluated their associations with Yale Global Tic Severity Scale (YGTSS) total scores. As shown in **Table 5**, multiple translated numerical signals were significantly associated with YGTSS total score across the full cohort.

**Table 5:** Correlation between subject-level translated numerical signals and YGTSS total score in the full cohort.

Signal category	Translated numerical signal	Pearson r	95% CI	p (FDR-corrected)	Spearman ρ	95% CI	p (FDR-corrected)
Physiological signals	Short-window spectral asymmetry index	0.36	(0.27, 0.44)	0.0004**	0.42	(0.34, 0.49)	<0.001**
	EDA mean amplitude ( μS)	0.33	(0.24, 0.41)	0.0009**	0.31	(0.22, 0.39)	0.0012**
Behavioral signals	Tic action unit confidence variance	0.54	(0.47, 0.60)	<0.001**	0.59	(0.53, 0.65)	<0.001**
	Abnormal motion energy peak (a.u.)	0.49	(0.41, 0.56)	<0.001**	0.51	(0.43, 0.58)	<0.001**
	Orofacial action unit activation strength	0.43	(0.34, 0.51)	<0.001**	0.46	(0.38, 0.54)	<0.001**

The short-window spectral asymmetry index was derived from low- and high-frequency components estimated within 20 s windows and should be interpreted only as a relative spectral descriptor, not as a standard LF/HF ratio or canonical HRV metric.

Among the physiological signals, the short-window spectral asymmetry index and EDA mean amplitude were both positively associated with YGTSS total score, indicating that the translated physiological descriptors varied with clinical severity. The behavioral signals showed stronger associations than the physiological signals. Specifically, tic action unit confidence variance, abnormal motion energy peak, and orofacial action unit activation strength showed moderate-to-strong positive correlations with YGTSS, suggesting that the translated visual descriptors captured clinically relevant variation in tic expression.

A similar severity-related pattern was observed across YGTSS-defined severity groups. As summarized in **Table 6**, all five translated numerical signals showed increasing trends from the mild group to the severe group, with significant Jonckheere–Terpstra trend tests and significant Kruskal–Wallis overall group effects. Because no *post-hoc* pairwise comparisons were performed, these findings are interpreted as evidence of overall group differences and monotonic severity-related trends, rather than pairwise differences between specific severity groups. Overall, the magnitude of change was larger for the behavioral signals than for the physiological signals, suggesting that translated visual descriptors captured severity-related variation more strongly, whereas physiological descriptors provided complementary contextual information.

**Table 6:** Severity-related trends in subject-level translated numerical signals.

Signal category	Translated numerical signal	Mild ( n=124) mean ± SD	Moderate ( n=163) mean ± SD	Severe ( n=130) mean ± SD	Trend p (J-T)	Group p (K-W)	Effect size ( $\eta^{2}$)
Physiological signals	Short-window spectral asymmetry index	1.22 ± 0.37	2.11 ± 0.49	3.06 ± 0.74	<0.001**	<0.001**	0.33
	EDA mean amplitude ( μS)	0.79 ± 0.23	1.46 ± 0.46	2.18 ± 0.54	<0.001**	<0.001**	0.37
Behavioral signals	Tic action unit confidence variance	0.09 ± 0.05	0.23 ± 0.08	0.36 ± 0.10	<0.001**	<0.001**	0.50
	Abnormal motion energy peak (a.u.)	15.4 ± 4.8	29.3 ± 6.0	46.9 ± 10.7	<0.001**	<0.001**	0.58
	Orofacial action unit activation strength	0.63 ± 0.16	1.21 ± 0.41	1.97 ± 0.57	<0.001**	<0.001**	0.45

Groups were defined by YGTSS severity, whereas table values are translated numerical signals. Values are subject-level medians across tic-positive windows, reported as mean ± SD within each group. J-T, Jonckheere–Terpstra trend test; K-W, Kruskal–Wallis test; η², effect size. No post-hoc pairwise comparisons were performed. The short-window spectral asymmetry index is not a standard LF/HF ratio or canonical HRV metric.

Taken together, these findings indicate that the translated numerical outputs preserved clinically meaningful subject-level structure. Although these associations should not be interpreted causally or used as diagnostic rules, they support the clinical relevance of the proposed evidence translation strategy.

### 3.5 Qualitative examples of translated evidence

**Figure 3:**
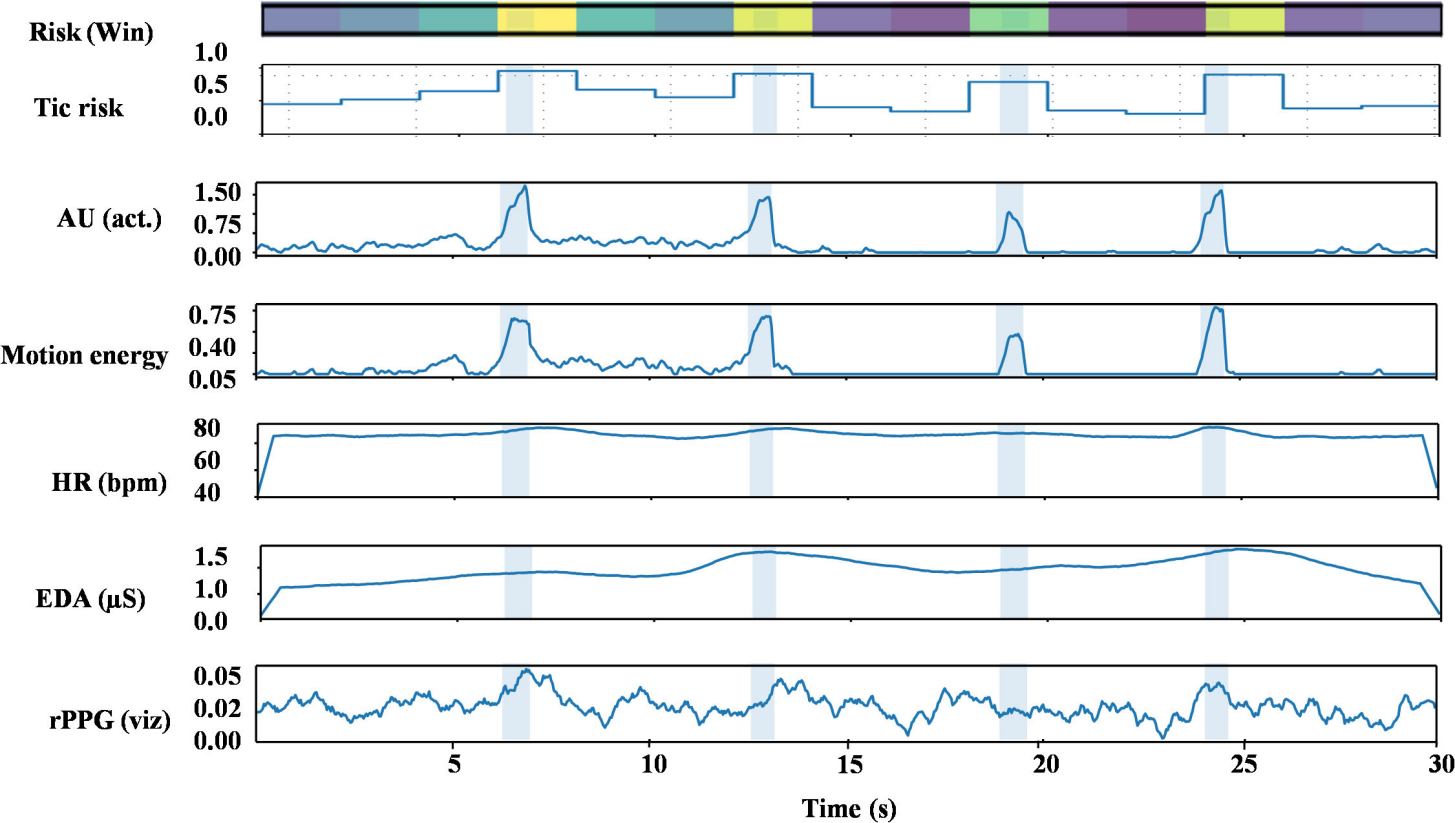
Time-aligned translated evidence generated by TIC-XNet. The curves show normalized tic risk, behavioral evidence, and physiological evidence along the same time axis.

Qualitative examples were used to illustrate how TIC-XNet organizes translated evidence across video, HR, and EDA modalities. As shown in **Figure 3**, the predicted tic probability, behavioral evidence, and physiological trajectories are displayed on a shared temporal axis. This layout allows direct inspection of whether risk increases occur at the same time as behavioral and physiological changes. In the illustrated examples, peaks in predicted tic risk were generally accompanied by localized behavioral evidence, such as increased orofacial activation or abnormal motion energy. In several windows, these behavioral peaks were also accompanied by concurrent HR- or EDA-related changes. This temporal correspondence suggests that TIC-XNet does not only output a window-level risk score, but also provides time-aligned multimodal evidence that can help users inspect when and why a tic-related decision is made.

**Figure 4:**
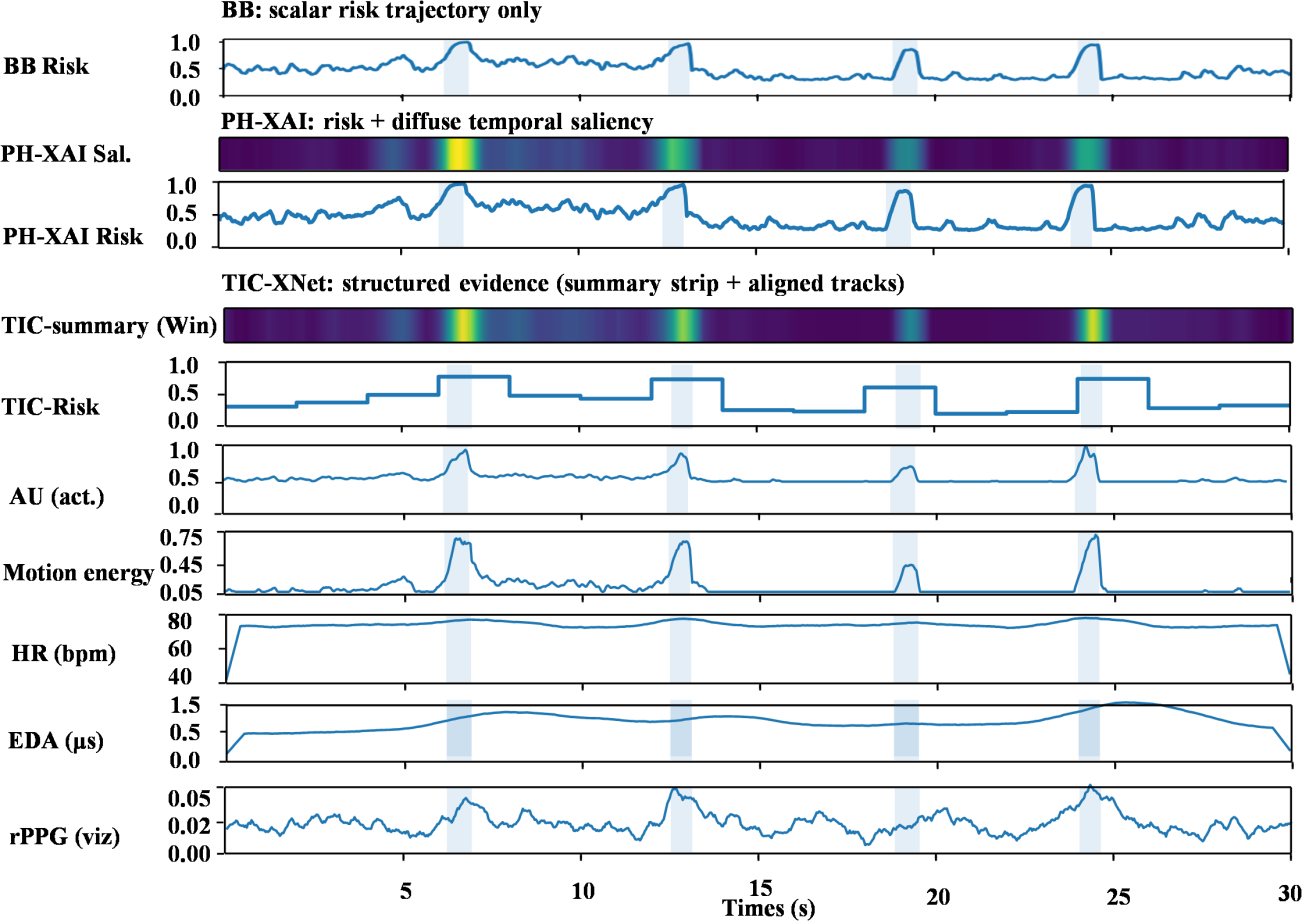
Qualitative comparison of output representations across BB, PH-XAI, and TIC-XNet. BB outputs scalar tic probabilities, PH-XAI provides *post-hoc* attribution maps, and TIC-XNet generates structured time-aligned evidence.

**Figure 4** further compares the output forms of the three model conditions. The black-box model provides only a scalar tic probability for each analysis window, which gives limited information about the temporal location or modality-specific basis of the decision. PH-XAI provides *post-hoc* saliency maps after prediction, but these outputs are less organized along a shared temporal structure and are therefore harder to interpret as event-level evidence. In contrast, TIC-XNet produces synchronized and structured evidence signals that indicate when tic risk increases and how video, HR, and EDA contribute to the decision. These qualitative examples illustrate the temporal clarity and organizational advantages of structured evidence translation, particularly for reviewing tic events that unfold over short time intervals.

## 4 Discussion

### 4.1 Principal findings

The present study systematically evaluated TIC-XNet as a multimodal framework for pediatric tic disorders designed to improve not only tic event detection, but also the interpretability, verifiability, and communicability of model-derived evidence. Across both structured clinical recordings and naturalistic home recordings, the results showed that explicitly organizing internal decision evidence into structured outputs was compatible with strong predictive performance. Under matched backbone settings, TIC-XNet achieved favorable window-level and event-level performance relative to both a prediction-only black-box model and a *post-hoc* explainable model. In addition, the model maintained low post-buffering prediction latency, indicating favorable computational efficiency once the current analysis window had been fully buffered. The smaller performance decline observed when moving from clinical to home recordings further suggests that structured cross-modal verification may improve robustness under more variable home-recording conditions.

Taken together, these findings suggest that, within the present experimental setting, embedding evidence organization directly into the model output specification was compatible with strong predictive performance and may support more stable performance across recording settings within the evaluated internal cohort. This robustness is clinically meaningful because tic disorders commonly show fluctuating manifestations across developmental stages and daily contexts (32). It is also consistent with evidence that tic severity is sensitive to contextual triggers, supporting the value of models that can remain stable across recording settings (33).

### 4.2 Explainability through evidence translation

Beyond detection accuracy, the main added value of TIC-XNet lies in the way evidence is incorporated into the prediction pathway itself. Rather than generating explanations only after prediction, the proposed framework derives temporally organized multimodal evidence from the same latent representations that support tic detection. This tighter coupling likely explains why TIC-XNet showed more faithful, more stable, and more temporally aligned explanations than the *post-hoc* baseline.

This distinction is important because clinical tic assessment is inherently time-resolved and depends on judging when an event occurs, how it unfolds, and what contextual features accompany it (34). Moreover, explainable machine learning methods may disagree substantially in the rationales they assign to the same prediction, which limits confidence in purely *post-hoc* interpretation (35). Recent work evaluating *post-hoc* explanations against transparent models has further shown that explanation quality cannot be assumed from visual plausibility alone (36). In healthcare AI, explainability is most valuable when the explanation design, evaluation strategy, and intended clinical use are explicitly aligned (37).

From this perspective, the present findings suggest that treating explainability as an explicit output objective, rather than as an after-the-fact visualization step, can produce evidence representations that are more testable, more reproducible, and more clinically coherent for time-resolved event detection tasks.

### 4.3 Association with clinical severity

The signal–severity analyses further suggest that the translated numerical outputs generated by TIC-XNet may retain clinically meaningful information beyond their immediate explanatory role. Because tic disorders fluctuate across time and context, subject-level summaries derived from synchronized behavioral and physiological recordings may provide quantitative descriptors of symptom expression that complement conventional clinical scales. The signal–severity findings are also clinically relevant in light of the heterogeneity reported in children newly diagnosed with tic disorders in China, where symptom profiles and management patterns vary substantially across patients (38).

In this context, the stronger severity-related pattern observed for the behavioral signals is clinically plausible, while the comparatively weaker physiological associations suggest a more supportive contextual role rather than a primary severity marker. These findings should not be interpreted causally, but they do indicate that the translated outputs were meaningfully related to independently assessed symptom burden.

Accordingly, the contribution of evidence translation may extend beyond explanation alone to clinically interpretable quantification, which may be useful for longitudinal review and context-sensitive monitoring.

### 4.4 Conceptual implications for explainability in tic disorders

At a conceptual level, TIC-XNet reframes explainability in tic disorder modeling from a *post-hoc* visualization problem to an evidence translation problem. More broadly, the proposed framework aligns with prior clinician-oriented visual analytics work showing that temporal model behavior becomes more interpretable when predictions are linked to inspectable evidence rather than displayed as isolated scores (39). It is also consistent with healthcare explanation research emphasizing the need to connect internal features back to concrete data-level evidence in order to support meaningful model review (40).

By contrast, TIC-XNet organizes internal model evidence into translated outputs that combine temporal evidence, region-level visual evidence, physiological support signals, numerical summaries, and deterministic textual descriptions within the same decision pathway. This design makes the reported outputs more directly traceable to the underlying model representations and therefore more amenable to systematic evaluation. In this sense, the central contribution of TIC-XNet is not merely that it “explains” predictions, but that it reorganizes internal evidence into forms that are more reviewable, more testable, and more clinically interpretable.

From this perspective, the proposed framework also suggests a broader methodological point: for time-resolved clinical AI tasks, the usefulness of explainability may depend less on how vividly an attribution map can be displayed and more on whether model-derived evidence can be translated into structured outputs that align with the way clinicians actually inspect, verify, and communicate events.

### 4.5 Clinical and translational implications

From a clinical perspective, this framework may help narrow the gap between model output and practical review. Tic disorders are commonly assessed during brief clinic visits, yet symptom expression often fluctuates across environments and over time. Time-aligned multimodal outputs derived from home or school recordings could allow clinicians to rapidly identify salient segments, inspect the dominant behavioral and physiological correlates, and contextualize symptom variability without relying exclusively on retrospective description. In this sense, evidence translation may improve the interpretability of longitudinal monitoring rather than merely improving classification performance.

The framework may also have value in clinician–family communication. Structured timelines that link observable behavior to synchronized physiological context can transform abstract model predictions or severity scores into concrete and reviewable episodes. This could support more transparent discussion of uncertainty, potential false positives, and context dependence during follow-up. From a deployment perspective, the observed low post-buffering prediction latency suggests that TIC-XNet may be computationally suitable for practical streaming monitoring scenarios once the current analysis window has been fully buffered. At the same time, runtime efficiency should be distinguished from temporal localization performance: low post-buffering prediction time does not by itself indicate immediate real-world alerting from true tic onset.

This is particularly relevant because tic-related urges and symptom structure vary across age groups, making longitudinal review more informative when it preserves developmental and contextual nuance (41). Prior video-based facial tic detection studies also suggest that behavior-only pipelines can identify visible events, but they do not by themselves provide the multimodal, time-aligned evidence needed for broader clinical review and communication (42).

Beyond immediate review utility, the observation that some translated evidence peaks occurred close to annotated tic onsets suggests that the model may capture temporally relevant multimodal changes around event emergence. However, these findings should be interpreted as evidence of fine-grained onset correspondence rather than as support for pre-onset warning. Future translational work should therefore assess computational efficiency, onset localization, and user-facing review workflows together under prospective monitoring conditions.

### 4.6 Limitations and future directions

Several limitations should be acknowledged.

First, although the study used a harmonized multimodal acquisition protocol across two clinical centers and included a relatively large pediatric tic cohort, the data were collected within a limited geographic region and were evaluated using an internal subject-level split rather than an independent external validation cohort. Accordingly, all performance claims in this study should be interpreted as internal-cohort findings. External generalizability across independent populations, acquisition devices, and clinical environments remains to be established through broader multi-center validation and prospective assessment beyond internal split performance (43).

Second, the signal–severity analyses should be interpreted as correlational rather than causal. The observed associations between translated numerical signals and YGTSS scores support the clinical relevance of the translated outputs, but they do not establish mechanistic links between individual behavioral or physiological descriptors and tic severity. These signals should therefore be viewed as clinically interpretable quantitative summaries rather than as surrogate biomarkers.

Third, the physiological interpretation of some translated outputs remains indirect. In particular, the short-window spectral asymmetry index was intentionally treated as a relative descriptor derived from 20 s windows rather than as a standard LF/HF ratio or canonical short-term HRV measure, because the 20 s analysis window is shorter than the duration typically recommended for standard frequency-domain HRV analysis. In addition, its physiological interpretation should remain cautious, because HRV-related estimation is sensitive to sensing modality and analytical conditions even at large scale (44). More broadly, wearable autonomic monitoring in real-life settings requires context-sensitive interpretation, since motion, environment, and daily activity can all affect physiological meaning near putative tic events (45).

Fourth, the reported runtime results reflect predictive computation after window buffering and should not be interpreted as end-to-end alerting delay from true tic onset to real-world notification. Likewise, the observed temporal proximity between fused evidence peaks and annotated onsets supports fine-grained within-window localization, but it does not imply pre-onset warning capability. Future deployment studies should jointly evaluate buffer design, alert timing, temporal localization accuracy, and user-facing review workflows under continuous monitoring conditions.

Fifth, the practical clinical utility of the translated outputs was evaluated only indirectly in the present study. Formal human-centered evaluation remains necessary to determine whether such explanations improve clinician trust, review efficiency, or communication quality in real decision-support settings (46).

Finally, the fairness-controlled comparison in this study was designed to isolate the added value of evidence translation under a matched predictive backbone. While this design strengthens internal interpretability comparisons, it does not exhaust the broader space of possible transparent, hybrid, or weakly structured explainable baselines. Future work should therefore compare TIC-XNet with a wider range of explainability paradigms while also testing external validation, prospective monitoring, and clinician-facing utility in real deployment settings.

## 5 Conclusion

This study presents TIC-XNet, a multimodal framework for pediatric tic disorder assessment that integrates evidence translation directly into the model output pathway. Using synchronized video and physiological signals, TIC-XNet jointly produces tic risk estimates and translated outputs, including temporally aligned evidence profiles, region-level visual evidence, physiological support signals, translated numerical signals, and deterministic textual summaries.

Across both structured clinical recordings and naturalistic home recordings, TIC-XNet achieved strong window-level and event-level detection performance relative to matched black-box and *post-hoc* explainable baselines. At the same time, the translated outputs showed higher decision fidelity, greater stability under perturbation, and closer temporal alignment with expert-annotated tic onsets. In addition, the subject-level translated numerical signals were systematically associated with YGTSS severity, supporting the clinical relevance of the proposed evidence translation strategy.

These findings indicate that evidence translation improves the interpretability and evaluability of multimodal tic assessment while remaining compatible with strong detection performance within the evaluated internal cohort. TIC-XNet provides a practical framework for transforming internal model evidence into structured and clinically reviewable outputs, with potential value for longitudinal monitoring and interpretable decision support in tic disorders.

## Data Availability

The original contributions presented in the study are included in the article/**Supplementary Material**. Further inquiries can be directed to the corresponding author.
