# Supplementary material for: TIC-XNet: a structured evidence translation framework for interpretable multimodal pediatric tic event detection with improved temporal alignment and fidelity
Source: Front Psychiatry. 2026 Jun 23;17:1862470. doi: 10.3389/fpsyt.2026.1862470 (PMC13337866; doi:10.3389/fpsyt.2026.1862470)
Supplement: Supplementary file 1 [file Supplementaryfile1.docx]

**Supplementary**

[Methods S1 Data preprocessing, windowing, and implementation details 1](#_Toc230373552)

[Methods S2 Self-supervised pretraining procedure 2](#_Toc230373553)

[Methods S3 Tic annotation protocol and inter-rater reliability assessment 2](#_Toc230373554)

[Methods S4 Fairness control and implementation details for comparator models 3](#_Toc230373555)

[Table S1. Video data acquisition and preprocessing settings 4](#_Toc230373556)

[Table S2. Physiological signal acquisition and preprocessing settings 5](#_Toc230373557)

[Table S3. Window segmentation and tic annotation settings 5](#_Toc230373558)

[Table S4. Model architecture and training hyperparameters for comparator models 6](#_Toc230373559)

[Table S5. TIC-XNet-specific architecture and parameter settings 8](#_Toc230373560)

# Methods S1 Data preprocessing, windowing, and implementation details

**S1.1 Temporal alignment and window segmentation**

All modalities were temporally aligned prior to model input. Video, heart rate (HR), and electrodermal activity (EDA) streams were mapped to a unified temporal axis using acquisition timestamps recorded during the same acquisition session. Continuous recordings were segmented into fixed-length analysis windows of 20 s with 10% overlap. Window boundaries were shared across video, HR, and EDA signals to ensure strict temporal correspondence among behavioral evidence, physiological dynamics, and tic-event labels.

Because video, HR, and EDA were acquired at different temporal resolutions, HR and EDA sequences were resampled onto a common temporal grid, while video streams were uniformly sampled or temporally pooled to the same number of temporal steps within each analysis window. Each window was therefore represented as aligned temporal steps for multimodal learning and within-window evidence localization.

**S1.2 Data preprocessing**

Video data were resized to 224 × 224 pixels and normalized prior to encoding. Video sequences were sampled at 15 frames per second, resulting in 300 frames per 20 s analysis window.

Physiological signals were filtered to reduce high-frequency noise and normalized at the subject level. HR artifacts were corrected using local thresholding, and short corrupted or missing segments were repaired using cubic spline interpolation. EDA signals were low-pass filtered and decomposed into tonic and phasic components when used for physiological feature extraction. HR and EDA signals were represented as fixed-length sequences at 10 Hz after preprocessing and resampling, resulting in 200 samples per 20 s analysis window for each physiological stream.

Windows with severe video loss, unrecoverable physiological artifacts, or incomplete timestamps were excluded according to predefined quality-control criteria.

**S1.3 Subject-level data splitting**

Data were split at the subject level into training, validation, and test sets at a ratio of 80%:10%:10% to prevent information leakage across windows from the same participant. Stratified sampling was used to preserve tic prevalence and recording-setting balance across splits. The same subject-level partitions were used across BB, PH-XAI, and TIC-XNet to ensure fair comparison.

**S1.4 Training configuration summary**

All models shared the same windowing strategy, preprocessing pipeline, subject-level data splits, training objective, and evaluation protocol. Training used weighted binary cross-entropy loss, the AdamW optimizer, an initial learning rate of $1\times10^{^{-}4}$, cosine annealing, batch size of 16, and up to 80 epochs. Early stopping was applied based on validation loss. Detailed architecture and hyperparameter settings are provided in Supplementary Methods S4 and Table S4.

# Methods S2 Self-supervised pretraining procedure

**S2.1 Rationale for self-supervised pretraining**

To mitigate the impact of limited labeled multimodal data on model training, self-supervised pretraining was performed prior to the main analysis. This pretraining stage aimed to learn generic spatiotemporal and physiological representations from publicly available multimodal datasets without using tic-related labels. The pretrained encoders were subsequently fine-tuned on the MindTS-MMD dataset for tic-event prediction.

**S2.2 Pretraining datasets**

Publicly available multimodal datasets containing synchronized video and physiological signals were used for pretraining. These datasets included recordings of human activities and affective states with aligned visual and physiological modalities. No subject overlap existed between the pretraining datasets and the MindTS-MMD cohort.

**S2.3 Pretraining strategy**

Self-supervised objectives were applied independently to each modality. For the video branch, contrastive learning was used to encourage temporally consistent representations across augmented views of the same video segment. For the physiological branch, including HR and EDA, temporal prediction and reconstruction objectives were employed to capture short-term dynamics and modality-specific temporal patterns.

Pretraining was conducted using the same encoder architectures later used in the main experiments, while prediction heads and clinical signal translation components were excluded during this stage.

**S2.4 Transfer to downstream task**

After pretraining, encoder weights were used to initialize the corresponding branches in all model conditions, including BB, PH-XAI, and TIC-XNet. Fine-tuning was performed end-to-end on the labeled MindTS-MMD dataset. No parameters from the pretraining stage were frozen during fine-tuning.

# Methods S3 Tic annotation protocol and inter-rater reliability assessment

**S3.1 Annotation protocol**

Tic events were annotated offline by raters with clinical experience in the assessment of tic disorders. Annotation was performed on synchronized multimodal recordings, with video and physiological signals presented in aligned timelines. Raters were instructed to identify tic events based on observable motor behaviors consistent with clinical definitions of tics.

For each tic event, annotators marked the onset time, offset time, and the primary behavioral manifestation, such as facial, upper-limb, or trunk-related movements. Annotations were performed continuously across the full duration of each recording session.

**S3.2 Annotation unit and temporal resolution**

Annotations were performed at the continuous time-series level rather than at the window level. Window-level labels used for model training and evaluation were subsequently derived by mapping annotated tic intervals onto predefined fixed-length analysis windows. A window was labeled as containing a tic event if any portion of an annotated tic interval overlapped with the window.

**S3.3 Inter-rater reliability assessment**

To assess annotation reliability, a subset of samples was independently annotated by two raters who were blinded to each other’s annotations. Inter-rater agreement was evaluated using metrics appropriate for temporal event annotation. Event-level annotation agreement was high, with Cohen’s κ = 0.86. Temporal consistency of tic onset and offset annotation was also strong, with ICC = 0.91.

Agreement metrics were computed based on temporal overlap between annotated tic intervals from different raters, with predefined tolerance thresholds for onset and offset alignment. These results confirmed the consistency and reproducibility of the annotation process.

**S3.4 Clinical severity assessment**

In addition to behavioral annotation, clinical tic severity was assessed using the Yale Global Tic Severity Scale (YGTSS), administered by trained clinicians. YGTSS scores were used exclusively for clinical characterization, stratified analyses, and signal–severity association analyses, and were not used as direct training targets for the predictive models.

# Methods S4 Fairness control and implementation details for comparator models

**S4.1 General fairness control principle**

To ensure that performance and interpretability differences across model conditions were attributable to explanation and evidence-translation paradigms rather than architectural or training confounds, strict fairness control was applied throughout model development and evaluation. All comparator models were designed under a shared multimodal learning framework, with key components and processing steps standardized wherever possible.

Specifically, fairness control focused on three aspects: (1) consistency of input representation and temporal windowing; (2) equivalence of backbone architecture and model capacity; and (3) alignment of training, validation, and testing protocols across model conditions.

**S4.2 Unified input processing and data split strategy**

All models operated on synchronized multimodal inputs consisting of video recordings and physiological signals segmented into fixed-length 20 s analysis windows with 10% overlap. Window length, temporal alignment strategy, resampling procedure, and window boundary definitions were kept consistent across models.

Subject-level data splitting was adopted to prevent information leakage across training, validation, and test sets. Data splits followed an identical 80%:10%:10% protocol across all model conditions, with the same subject assignments used for training, validation, and testing. This ensured that all models were evaluated on exactly the same data samples under comparable conditions.

**S4.3 Shared backbone architecture and model capacity**

The black-box model (BB), post-hoc explainable model (PH-XAI), and TIC-XNet shared the same core backbone architectures for modality-specific feature extraction, multimodal fusion, and prediction. This included identical video encoders, physiological signal encoders, fusion mechanisms, and prediction head structures.

The video branch used a 3D-ResNet-18 encoder to extract spatiotemporal visual features from uniformly sampled frame sequences. The physiological branches used 1D-CNN + GRU encoders to model short-term HR and EDA dynamics. The physiological GRU hidden size was 128. Video and physiological feature dimensions were projected to 256 and concatenated into a 512-dimensional fused representation. The prediction head was a two-layer MLP with 128 hidden units and a sigmoid output for window-level tic/non-tic classification.

Model capacity, including number of layers, hidden dimensions, feature embedding sizes, and fusion dimensionality, was kept equivalent across model conditions wherever applicable. PH-XAI generated explanations only at inference and introduced no additional trainable parameters.

**S4.4 Training protocol and optimization consistency**

All models were trained using the same loss function, optimization framework, batch size, and training schedule. Weighted binary cross-entropy loss was used for window-level tic-event classification. Training was conducted using the AdamW optimizer with an initial learning rate of 1 × 10^-4, cosine annealing learning-rate scheduling, batch size of 16, and up to 80 epochs. Early stopping was applied based on validation loss.

The probability threshold used for binarizing window-level predictions was selected on the validation set by maximizing window-level F1 and was then fixed for all held-out test evaluations. These training and thresholding procedures were kept identical across BB, PH-XAI, and TIC-XNet.

**S4.5 Controlled differences across model conditions**

Differences between comparator models were intentionally limited and explicitly controlled. The black-box model (BB) produced only window-level tic risk predictions without any explanatory outputs. The post-hoc explainable model (PH-XAI) used the same trained prediction model as BB and generated attribution-based explanations only at inference time, including spatiotemporal Grad-CAM for video and Integrated Gradients for physiological signals, without modifying model parameters or training dynamics.

TIC-XNet shared the same predictive backbone and multimodal input pipeline but additionally translated internal decision evidence into structured visual, numerical, and textual outputs. Thus, the only intentional difference among model conditions was the explainability paradigm.

**S4.6 Summary**

Through unified input processing, shared backbone architectures, aligned training protocols, identical subject-level partitions, and constrained implementation differences, this study ensured that observed differences in interpretability, stability, and clinical usability were attributable primarily to explanation and evidence-translation strategies rather than confounding implementation factors.

# Table S1. Video data acquisition and preprocessing settings

| **Category** | **Parameter** | **Specification** |
| --- | --- | --- |
| Recording content | Target region | Face and upper body |
| Recording content | Recording scenario | Clinical assessment / home environment |
| Recording content | Sensor type | RGB camera |
| Camera | Input resolution | 224 × 224 after resizing |
| Camera | Frame rate | 15 fps after uniform sampling |
| Camera | Color format | RGB |
| Preprocessing | Spatial normalization | Resizing and intensity normalization |
| Preprocessing | Temporal processing | Fixed-length frame sequence extraction per 20 s window |
| Preprocessing | Region handling | Full-frame input without landmark-based cropping |
| Output | Video representation | 300-frame sequence per 20 s analysis window |

***Note.*** *Table S1 summarizes acquisition and preprocessing parameters for the video modality. Video recordings focused on facial and upper-body regions to capture tic-related motor behaviors. Video streams were uniformly sampled at 15 fps after preprocessing, and each 20 s window was represented as a 300-frame sequence.*

# Table S2. Physiological signal acquisition and preprocessing settings

| **Signal** | **Parameter** | **Specification** |
| --- | --- | --- |
| Heart rate (HR) | Sensor type | Wearable heart-rate sensor |
| Heart rate (HR) | Signal type | Time-domain HR/IBI-related sequence |
| Heart rate (HR) | Temporal representation | 10 Hz sequence after preprocessing and resampling |
| Heart rate (HR) | Preprocessing | Noise filtering, local threshold artifact correction, cubic spline interpolation, and subject-level normalization |
| Electrodermal activity (EDA) | Sensor type | Wearable EDA sensor |
| Electrodermal activity (EDA) | Signal components | Tonic and phasic activity |
| Electrodermal activity (EDA) | Temporal representation | 10 Hz sequence after preprocessing and resampling |
| Electrodermal activity (EDA) | Preprocessing | Low-pass filtering, artifact removal, smoothing, and subject-level normalization |
| Synchronization | Alignment strategy | Timestamp-based alignment within each acquisition session |
| Output | Physiological representation | Fixed-length HR and EDA sequences with 200 samples per 20 s window |

***Note.*** *Table S2 summarizes acquisition and preprocessing parameters for physiological signals. HR and EDA streams were mapped to a unified temporal axis and resampled to 10 Hz after preprocessing, yielding 200 samples per 20 s analysis window for each physiological modality.*

# Table S3. Window segmentation and tic annotation settings

| **Category** | **Parameter** | **Specification** |
| --- | --- | --- |
| Windowing | Window length | 20 s |
| Windowing | Window overlap | 10% |
| Windowing | Boundary alignment | Identical across video, HR, and EDA modalities |
| Windowing | Temporal representation | Aligned temporal steps within each analysis window |
| Annotation | Annotation mode | Offline manual annotation |
| Annotation | Annotators | Clinically experienced raters |
| Annotation | Annotation unit | Continuous tic interval |
| Annotation | Labeled attributes | Tic onset, offset, and primary behavioral manifestation |
| Label assignment | Positive-window definition | Any overlap with an expert-annotated tic interval |
| Reliability | Dual annotation | Subset of independently annotated samples |
| Reliability | Event-level agreement | Cohen’s κ = 0.86 |
| Reliability | Temporal consistency | ICC = 0.91 for tic onset/offset annotation |
| Clinical assessment | Tic severity scale | Yale Global Tic Severity Scale (YGTSS) |
| Usage | Downstream analysis | Model training, evaluation, and clinical relevance analysis |

***Note.*** *Table S3 summarizes the window segmentation and tic annotation settings. Tic events were manually annotated with onset and offset timing, and window-level labels were derived by projecting annotated tic intervals onto aligned 20 s analysis windows.*

# Table S4. Model architecture and training hyperparameters for comparator models

| **Category** | **Parameter** | **BB (Black-box)** | **PH-XAI (Post-hoc explainable)** |
| --- | --- | --- | --- |
| Input and windowing | Window length | 20 s | 20 s |
| Input and windowing | Window overlap | 10% | 10% |
| Input and windowing | Video frames per window | 300 (15 fps × 20 s) | 300 (15 fps × 20 s) |
| Input and windowing | HR/EDA samples per window | 200 (10 Hz × 20 s) | 200 (10 Hz × 20 s) |
| Video encoder | Backbone | 3D-ResNet-18 | 3D-ResNet-18 |
| Video encoder | Input resolution | 224 × 224 | 224 × 224 |
| Video encoder | Pretraining | Self-supervised video pretraining | Self-supervised video pretraining |
| Physiological encoder | Encoder type | 1D-CNN + GRU | 1D-CNN + GRU |
| Physiological encoder | CNN kernel size | 5 | 5 |
| Physiological encoder | GRU hidden units | 128 | 128 |
| Physiological encoder | HR normalization | Z-score per subject | Z-score per subject |
| Physiological encoder | EDA preprocessing | Filtering and tonic–phasic decomposition | Filtering and tonic–phasic decomposition |
| Multimodal fusion | Fusion strategy | Feature concatenation | Feature concatenation |
| Multimodal fusion | Fusion dimension | 512 | 512 |
| Prediction head | Head type | Two-layer MLP | Two-layer MLP |
| Prediction head | Hidden units | 128 | 128 |
| Prediction head | Output activation | Sigmoid | Sigmoid |
| Prediction head | Prediction target | Tic vs. non-tic | Tic vs. non-tic |
| Training | Loss function | Weighted binary cross-entropy | Weighted binary cross-entropy |
| Training | Optimizer | AdamW | AdamW |
| Training | Initial learning rate | 1 × 10^-4 | 1 × 10^-4 |
| Training | Learning-rate schedule | Cosine annealing | Cosine annealing |
| Training | Weight decay | 1 × 10^-5 | 1 × 10^-5 |
| Training | Batch size | 16 | 16 |
| Training | Maximum epochs | 80 | 80 |
| Training | Early stopping | Validation loss | Validation loss |
| Data split | Split strategy | Subject-level | Subject-level |
| Data split | Train / validation / test | 80 / 10 / 10 | 80 / 10 / 10 |
| Explainability | Video explanation | None | Spatiotemporal Grad-CAM |
| Explainability | Physiological explanation | None | Integrated Gradients |
| Explainability | Explanation stage | N/A | Inference only |
| Explainability | Trainable parameters added | None | None |

***Note.*** *Table S4 summarizes model architecture and training hyperparameters for the comparator models. BB and PH-XAI shared the same core predictive backbone, fusion strategy, prediction objective, data split protocol, and training configuration. PH-XAI generated explanations only at inference and did not introduce additional trainable parameters.*

# Table S5. TIC-XNet-specific architecture and parameter settings

| **Category** | **Component** | **Parameter** | **Setting** | **Description** |
| --- | --- | --- | --- | --- |
| Input and windowing | Analysis window | Window length | 20 s | Same window length as comparator models. |
| Input and windowing | Analysis window | Window overlap | 10% | Used to improve temporal continuity without excessive redundancy. |
| Input and windowing | Video input | Frames per window | 300 | 15 fps × 20 s. |
| Input and windowing | Physiological input | Samples per window | 200 | 10 Hz × 20 s for HR and EDA after preprocessing and resampling. |
| Modality encoders | Video encoder | Backbone | 3D-ResNet-18 | Shared with BB and PH-XAI. |
| Modality encoders | Video encoder | Output dimension | 256 | Window-level video feature dimension. |
| Modality encoders | Physiological encoder | Architecture | 1D-CNN + GRU | Shared base physiological encoder. |
| Modality encoders | Physiological encoder | GRU hidden units | 128 | Models short-term physiological dynamics. |
| Modality encoders | Physiological encoder | Output dimension | 256 | Physiological feature dimension after temporal aggregation. |
| Multimodal fusion | Fusion module | Fusion strategy | Feature concatenation | Concatenates video and physiological features. |
| Multimodal fusion | Fusion module | Fused dimension | 512 | Input to prediction and evidence heads. |
| Prediction head | Risk predictor | Architecture | Two-layer MLP | Hidden layer followed by sigmoid output. |
| Prediction head | Risk predictor | Hidden units | 128 | ReLU activation. |
| Prediction head | Risk predictor | Output | 1 | Window-level tic probability. |
| Decision evidence heads | Visual evidence head | Input | Video and fused features | Uses modality-specific and fused information. |
| Decision evidence heads | Visual evidence head | Hidden units | 128 | Evidence projection layer. |
| Decision evidence heads | Visual evidence head | Output dimension | 64 | Window-level visual evidence vector. |
| Decision evidence heads | Visual evidence head | Normalization | L2 normalization | Stabilizes evidence magnitude across windows. |
| Decision evidence heads | Physiological evidence head | Input | Physiological and fused features | Uses physiological and fused information. |
| Decision evidence heads | Physiological evidence head | Hidden units | 96 | Reduced capacity reflecting lower-dimensional physiological input. |
| Decision evidence heads | Physiological evidence head | Output dimension | 32 | Window-level physiological evidence vector. |
| Decision evidence heads | Physiological evidence head | Normalization | L2 normalization | Same normalization strategy as visual evidence. |
| Evidence usage | Evidence scope | Temporal unit | Window-level | No frame-level or sample-level supervision was used. |
| Evidence usage | Trainability | Evidence heads | Jointly trained | Evidence heads were trained end-to-end with the prediction task. |
| Clinical signal interface | Evidence-to-signal mapping | Translation mechanism | Rule-based, non-generative | Structured evidence outputs were produced using deterministic rules. |
| Clinical signal interface | Text generation | Output type | Template-based textual summary | No generative language model was used. |

***Note.*** *Table S5 summarizes TIC-XNet-specific architecture and parameter settings. TIC-XNet extends the shared predictive backbone by adding structured evidence heads and deterministic evidence-to-signal translation. These components generate visual, numerical, and textual outputs while preserving the same input processing, backbone, fusion scheme, prediction task, and training protocol used by the comparator models.*
